# Air caloric test in canal wall down mastoidectomy

**DOI:** 10.1590/S1808-86942012000300004

**Published:** 2015-10-14

**Authors:** Lucia Kazuko Nishino, Lidio Granato

**Affiliations:** a MSc in Health Sciences at Faculdade de Ciências Médicas da Santa Casa de São Paulo (Speech and hearing therapist, supervisor in the audiology specialization program at Irmandade da Santa Casa de Misericórdia de São Paulo).; b PhD in otorhinolaryngology at Escola Paulista de Medicina (Professor at Faculdade de Ciências Médicas da Santa Casa de São Paulo). Faculdade de Ciência Médicas da Santa Casa de São Paulo Irmandade da Santa Casa de Misericórdia de São Paulo.

**Keywords:** caloric tests, ear, electronystagmography, mastoid, middle, nystagmus, physiologic

## Abstract

Since the 1970s, few studies have been conducted to elucidate the use of caloric tests on middle ear disorders, despite the many controversies that this test may produce in anatomical structures that are so distinct. In cases of mastoidectomy, such studies are even rarer.

**Objective:**

This study aims to analyze the findings from air caloric stimulation done in individuals submitted to unilateral radical mastoidectomy without complaints of dizziness.

**Materials and Method:**

Thirty-six individuals without vestibular complaints were enrolled in this prospective study. Air caloric stimulation was offered to all subjects. Twenty-one individuals had undergone unilateral open mastoidectomy and 15 did not present any middle or outer ear abnormalities.

**Results:**

80.95% of the individuals presented asymmetrical responses in the cold caloric test, with greater response on the side of the open mastectomy. In 72.73% of the subjects the same effect was observed in the hot caloric test. The four stimulation modes revealed asymmetries in both hot and cold tests in 81.82% of the cases. Paradoxical stimulation was observed in 47.61% of hot caloric tests.

**Conclusion:**

Nystagmic responses on the side of the open mastoidectomy were greater than on the healthy side. Paradoxical stimulation in caloric tests was a frequent finding. No hypofunctioning responses were found.

## INTRODUCTION

Air is gradually replacing water in caloric tests. Many are the reasons for air to be chosen over water. Among them are the possibility of testing individuals with middle and outer ear disorders, the fact that air is technically easier to handle than water, and increased comfort levels experienced by test subjects^1^, ^2^, ^3^, ^4^, ^5^, ^6^, ^7^, ^8^, ^9^, ^10^.

Dizziness is a symptom experienced by 10% of the world's population^11^, and otitis media is a highly prevalent condition worldwide^12^, ^13^. Therefore, it is no wonder these symptoms may occur concurrently even whey they are unrelated. In post-operative middle ear complications this combination of symptoms may occur due to the manipulation of the auditory vestibular apparatus^14^, ^15^, ^16^. Iatrogenic complications arising from mastoidectomy and tympanomastoidectomy may lead to vestibular injuries secondary to direct trauma or subsequent infection^17^.

Caloric tests offer significant vestibular information, as each labyrinth is examined separately and valuable data is gathered on the topographic diagnosis of dizziness^1^, ^2^, ^3^, ^4^, ^5^.

Awareness levels over the possibility of performing caloric tests in individuals with middle ear disorders have increased, and so have the number or orders for this test. One should yet be reminded that middle ear disorders may hamper the interpretation of caloric test results and produce controversial readings^1^, ^9^, ^18^, ^19^, ^20^.

There is only one reference in the literature on the assessment of the vestibular system of ears submitted to unilateral canal wall down (CWD) mastoidectomy with the purpose of offering information on labyrinthine function during caloric stimulation in anatomically altered subjects. However, the number of cases is limited and do not allow for proper analysis of caloric test findings^18^.

Hence the need to verify the possibility of establishing reliable parameters to perform caloric tests in individuals with altered ear anatomy, as such parameters may be different from the ones applied to individuals with preserved ear anatomy^21^.

In order for the assessment of labyrinthine diseases that may occur in individuals with anatomically different ears not to cast doubt upon the results of caloric tests, the authors of this study decided to look into air caloric stimulation in unilateral CWD mastoidectomy patients without complaints of dizziness to establish reliable parameters.

This paper aims to analyze the findings of air caloric tests done in unilateral canal wall down mastoidectomy patients without complaints of dizziness.

## MATERIALS AND METHOD

This study was approved by the Research Ethics Committee of our institution and granted permit number 155/09. This is a contemporary cross-sectional cohort study. Twenty-one unilateral canal wall down mastoidectomy patients without dizziness were enrolled (6 males and 15 females). The same set of tests was performed in 15 healthy controls (8 males and 7 females). The enrolled subjects were split into two groups: a control group containing 15 healthy individuals without middle ear disorders or dizziness; and a case group made up of 21 unilateral CWD mastoidectomy patients without dizziness.

All participants were given clarifications on the goals of the study and invited to join it. They were enrolled only after signing an informed consent form.

Forty-nine unilateral CWD mastoidectomy patients were interviewed and submitted to pure-tone and speech audiometry tests to check whether they could be enrolled in the trial. Twenty-eight (57.14%) subjects were excluded for having severe or profound sensorineural hearing loss, diseases or obstructions in the ear canal opposite to the side in which CWD mastoidectomy was performed, presence of spontaneous nystagmus with open or closed eyes, or dizziness.

Basic audiological examination was performed using a two-channel Itera Madsen. Tests included pure-tone audiometry, speech recognition threshold (SRT), and speech detection threshold (SDT) tests.

Vestibular evaluation was performed using a device made by VENG Digital Neurograff Eletromedicina Ind. & Com. Ltda, a specific software program, and a lamp to present visual cues. Caloric stimulation was done using an NGR05 ear calorimeter from the same maker. The cutoff values defining normal results were the ones present in the specific software program, along with the corrections proposed by Costa et al.^8^.

The vestibular test set included positional nystagmus, eye motion calibration, spontaneous nystagmus with open and closed eyes, and air caloric tests at 42ºC and 18ºC in 80-second cycles.

Test results were recorded and treated in a computer to produce data on gain, latency, precision, and slow phase velocity (SPV), besides all the calculation required for each test. The formula proposed by Jongkees was used in the calculation of the four stimulation events considering 33% for LP and 22% for DP.

SPV values in hot and cold tests were calculated separately. Mean values (maximum value minus minimum value; the outcome is then divided by the sum of maximum and minimum values and multiplied by 100) were used to calculate directional preponderances in the cold and hot tests. Symmetry was defined for values under 30% and asymmetry for values above 30%.

Calculations were done for both groups. CWD mastoidectomy patients (21 ears) were compared to all other ears without middle ear alterations (51 ears; 30 from the control group and 21 from the case group).

Audiological and vestibular tests were done on the same day.

Paradoxical stimulation events in hot caloric tests were excluded from the isolated calculation of directional preponderances in hot caloric tests and in the four stimulation events.

Selected subjects were advised to avoid smoking and taking non-essential drugs such as tranquilizers and to stop eating three hours before the tests.

Software EPI-INFO^®^ release 3.3.2 was used in data statistical treatment. Fisher's exact test was used to analyze nonparametric data. The chi-square test was used for parametric data and analyses of mean values and standard deviations. Statistical significance was attributed when *p* < 0.05. Asterisks were placed beside the *p* value in all statistically significant results.

## RESULTS

Fisher's exact test did not find any statistically significant differences concerning the gender of case and control group members (*p* = 0.1333).

Age did not elicit statistically significant differences in the chi-square test (*p* = 1.00). Mean ages were 36.73 ± 11.25 in the control group and 37.19 ± 14.30 in the case group.

Caloric tests were analyzed separately for each stimulation temperature. When right ears were compared to left ears in cold stimulation, the case group had 17/21 (80.95%) asymmetric responses (values above 30%) and 4/21 (19.05%) symmetric responses (values under 30%). Asymmetric responses were not observed in the control group.

The mean values in cold stimulation events were analyzed for both groups. The chi-square test elicited a statistically significant difference between the groups (*p* < 0.001*) (Table 1).

**Table 1:** Mean nystagmus SPV in cold stimulation of all groups.

	Mean SPV	Standard Deviation	Min. SPV	Max. SPV
Control Group	12.16	6.36	2.37	28.64
Case Group	49.41	21.87	10.74	80.79

SPV = slow phase velocity *p* < 0.001*.

The mean value of all cold stimulation events included 72 ears, 51 of which with preserved eardrums (21 ears from case group subject and 30 from control group individuals).

The mean values in cold stimulation events considering 72 ears in the intact eardrum and CWD mastoidectomy groups were analyzed. The chi-square test elicited a statistically significant difference between the groups (*p* < 0.001*) (Table 2).

**Table 2:** Mean SPV in cold stimulation events for ears with intact eardrums and CWD mastoidectomy.

	Ears	Mean SPV	Standard Deviation	Min. SPV	Max. SPV
Intact eardrums	51	11.34	4.92	3.80	32.10
CWD mastoidectomy	21	27.57	14.51	5.80	66.50

SPV = slow phase velocity *p* < 0.001*.

Ten subjects were excluded from the analysis of hot stimulation events as they experienced paradoxical stimulation in the hot caloric test. Twenty-six subjects were included, 15 from the control and 11 from the case group. Symmetric responses were found for 15/15 (100%) subjects in the control group and 3/11 (27.27%) individuals in the case group. Asymmetric responses were not recorded in the control group, whereas in the case group they were found for 8/11(72.73%) individuals.

The mean values in hot stimulation events considering 62 ears in the intact eardrum and CWD mastoidectomy groups were analyzed. The chi-square test elicited a statistically significant difference between the groups (*p* < 0.001*) (Table 3).

**Table 3:** Mean nystagmus SPV during hot stimulation of all ears with intact eardrum and submitted to CWD mastoidectomy, minus individuals with paradoxical stimulation.

	Ears	Mean SPV	Standard Deviation	Min. SPV	Max. SPV
Intact eardrum	51	7.46	2.45	2.80	15.40
CWD mastoidectomy	11	21.90	11.45	6.40	44.40

SPV = slow phase velocity *p* < 0.001*.

Four stimulation events were performed in 26 subjects, while 10 were excluded due to paradoxical responses in the hot caloric test.

Jongkees' formula was used to analyze the data from the four stimulation events. In the case group 9/11 (81.82%) individuals had altered responses (values above 33%) and 2/11 (18.18%) had unaltered responses (values under 33%).

The mean values of the four stimulation events calculated using Jongkees' formula were calculated for the case and control groups. he chi-square test elicited a statistically significant difference between the groups (*p* < 0.001*) (Table 4).

**Table 4:** Mean values for four stimulation events calculated based on Jongkees' formula, minus subjects presenting paradoxical responses in the hot caloric test.

	Ears	Mean SPV	Standard Deviation	Min. SPV	Max. SPV
Intact eardrum	15	11.65	5.35	2.99	22.31
CWD mastoidectomy	11	49.98	22.85	9.56	77.57

SPV = slow phase velocity *p* < 0.001*.

Ten (47.61%) subjects in the case group had paradoxical responses in the hot caloric test, a statistically significant event (*p* = 0.0014*) according to the chi-square test.

## DISCUSSION

In the 1970s a few studies ere initiated with the purpose of performing air caloric tests n subjects with compromised eardrums^2^, ^3^, ^10^, ^18^. Given the criticism on the inconsistent responses produced by air stimulators, several studies were carried out to prove the effectiveness and reliability of stimulation^4^, ^5^, ^6^, ^8^. Despite the proven efficacy of air stimulation, only a few papers have been published on the topic. In 2009, Nishino et al.^9^ carried out a study on air caloric stimulation in subjects with unilateral perforated eardrums and verified the viability of this test mode in this patient group.

The analyses performed in this study to verify age and gender of group members were done solely with the purpose of minimizing the bias that would be introduced if they were not similar.

In cold stimulation, 80.95% of the stimuli produced asymmetric values, as also seen in studies that reported exacerbated responses in CWD mastoidectomy patients^3^, ^18^.

More variability was observed in responses to cold stimulation, as verified in the standard deviation recorded for the case group. Such variation may relate to the probably different anatomies that resulted from surgical manipulation^21^.

The mean nystagmic response in the cold stimulation of ears with intact eardrums was 12.16°/s, while the mean nystagmic response in CWD mastoidectomy ears was four times greater (49.41°/s). This is related to the absence of structures in the open cavity. There are no obstacles to air flow and stimulation is more intense, as reflected in the exacerbated responses observed when compared to the middle ear of the unaffected side. This finding is in agreement with a study in which the author discusses that equal stimulation in different cavities may produce different responses in the caloric test. However this study only alluded to this possibility, and failed to support it with data^21^.

The same exacerbated responses seen in cold caloric stimulation were also seen in hot stimulation, proving that greater nystagmic responses are related to the characteristics of the open cavity rather than temperature^3^, ^18^.

As in Nishino et al.^9^, paradoxical stimulation in the hot caloric test was a frequent finding (47.61%). This phenomenon occurred due to the presence of fluids in the open cavity as seen in perforated eardrums, i.e., nystagmus was observed in the opposite side to what had been expected in the hot caloric test as the fluid evaporated inside the cavity, thus producing cold instead of hot stimulation. Other authors described it as a common phenomenon^1^, ^3^, ^18^, ^19^.

The presence of different anatomical structures during caloric stimulation poses a few hurdles in obtaining reliable responses to allow for the comparison of labyrinths separately - one of the advantages of bithermal caloric tests. Despite the difficulty inherent to stimulating labyrinths through different cavities, given specific size, epithelium, and anatomy characteristics, this study este estudo nos trouxe algumas considerações importantes. In most cases the nystagmic responses on the side of the CWD mastoidectomy had greater amplitude than the results of the unaltered side. Therefore, patients suspected for labyrinthine injury on the CWD mastoidectomy side may be suggestive of vestibular injury if the caloric test has diminished responses on this side, once our study did not reveal hyporeflexive or mild nystagmus on the CWD mastoidectomy side. On the other hand, increased nystagmic responses on the CWD mastoidectomy side may not be regarded as labyrinthine disease or consider labyrinthine preponderance to the side of CWD mastoidectomy to infer the presence of deficit dysfunction on the other side.

Air caloric tests were not studied for years in middle ear disorders. Air caloric stimulators are more accurate and popular today, and many are skilled at performing precise air caloric tests. These changes have led many physicians to order this vestibular test for many of their patients, including the ones with middle ear alterations. Many questions sprang from the wider use of this test, thus adding to the relevance of studies developed to assess the test's effectiveness and diagnostic accuracy.

## CONCLUSION

Nystagmic responses on the CWD mastoidectomy side were greater when compared to responses from the healthy ear. Labyrinthine preponderance (LP) was observed on the CWD mastoidectomy side. Paradoxical stimulation in hot caloric tests was a frequent finding. Hypofunctional responses were not found in any of the stimulation events, mainly on the CWD mastoidectomy side.

## References

[1] Aantaa E (1966). Caloric Test with air. Preliminary Report. Acta Otolaryngol..

[2] Albernaz PL, Ganança MM (1972). The use of air in vestibular caloric stimulation. Laryngoscope..

[3] Barber HO, Harmand WM, Money KE (1978). Air caloric stimulation with tympanic membrane perforation. Laryngoscope..

[4] Ford CR, Stockwell CW (1978). Reliabilities of air and water caloric responses. Arch Otolaryngol..

[5] Tole JR (1979). A protocol for the air caloric test and a comparasion with a standard water caloric test. Arch Otolaryngol..

[6] Gao YZ, Sze YY, Shen L (1983). The air caloric test and its normal values. Adv Otorhinolaryngol..

[7] Ganança CF, Souza JAC, Segatin LA, Caovilla HH, Ganança MM (2000). Limites normais dos parâmetros de avaliação à vectonistagmografia digital Neurograff. Acta Awho..

[8] Costa KCF, Silva SMR, Ganança CF (2005). Estudo das provas oculomotoras e vestibulares por meio da vectonistagmografia digital. Disturb Comun..

[9] Nishino LK, Granato L, Taguchi CK (2009). Air stimulation in tympanic perforation: inverted nystagmus study. Braz J Otorhinolaryngol..

[10] Kozie DW, Hassul M, Kimm J (1976). Effect of tympanic membrane perforations on air caloric response in monkeys. Trans Sect Otolaryngol Am Acad Ophthalmol Otolaryngol..

[11] Ganança MM, Caovilla HH, Ganança MM (1998). Vertigem tem cura?.

[12] Brown OE, Meyerhoff WL (1988). Complication and sequelae of chronic suppurative otitis media. Ann Otol Rhinol Laryngol..

[13] Scheibe AB, Smith MM, Schmidt LP, Schmidt VB, Dornelles C, Carvalhal LHSK (2002). Estudo da orelha contralateral na otite média crônica: “Efeito Orloff^®^”. Rev Bras Otorrinolaringol..

[14] Dawes PJ (1999). Early complications of surgery for chronic otitis media. J. Laryngol Otol..

[15] Pickuth D, Brandt S, Berghaus A, Spielmann RP, Heywang-Köbrunner SH (2000). Vertigo after stapes surgery: the role of high resolution. CT Br J Radiol..

[16] Atacan E, Sennaroglu L, Genc A, Kaya S (2001). Benign paroxysmal positional vertigo after stapedectomy. Laryngoscope..

[17] Portman D, Rezende Ferreira D (2003). Delayed labyrinthine fistula in canal wall down mastoidectomy. Rev Laryngol Otol Rhinol (Bord)..

[18] Paparella MM, Rybak L, Meyerhoff WL (1979). Air caloric testing in otitis media. (preliminary studies). Laryngoscope..

[19] Norré ME, Renier B (1979). Inverted caloric nystagmus by warm air stimulation. Acta Otorhinolaryngol Belg..

[20] Coles RR, Snashall SE (1973). False negative response from caloric stimulation. Acta Otolaryngol..

[21] Mckenzie W (1949). Vertigo after radical mastoidectomy. J Laryngol Otol..

